# Cornea Verticillata in classical Fabry disease, first from Sri Lanka: a case report

**DOI:** 10.1186/s12887-020-02237-z

**Published:** 2020-07-08

**Authors:** Hasani Hewavitharana, Eresha Jasinge, Hiranya Abeysekera, Jithangi Wanigasinghe

**Affiliations:** 1 Professorial Paediatric Unit, Lady Ridgeway Hospital for Children, Colombo 08, Sri Lanka; 2 Department of Chemical Pathology, Lady Ridgeway Hospital for Children, Colombo 08, Sri Lanka; 3 Department of Paediatric Ophthalmology, Lady Ridgeway Hospital for Children, Colombo 08, Sri Lanka; 4 Department of Paediatrics, Faculty of Medicine, University of Colombo, Colombo 8, Sri Lanka

**Keywords:** Fabry disease, α-Galactosidase A, Cornea Verticillata, *GLA* gene, Case report

## Abstract

**Background:**

Fabry disease is a rare inborn error of metabolism with profound clinical consequences if untreated. It is caused by the deficiency of α galactosidase A enzyme and is the only lysosomal storage disorder with an X linked inheritance. Confirmation requires genetic analysis of Galactosidase Alpha (GLA) Gene, which is often a challenge in resource-poor settings. Despite these technological limitations, specific clinical features in this condition can establish the diagnosis.

**Case presentation:**

We report on a 13-year old male who presented with an afebrile convulsion with a background history of chronic burning sensation of hands and feet and anhidrosis for 2 years duration with a similar history of episodic acroparesthesia in the other male sibling. The early clinical diagnosis was based on the history and detection of Cornea Verticillata on eye examination. Biochemical confirmation was established with detection of low α galactosidase A enzyme levels and a missense mutation of the Galactosidase Alpha (*GLA*) Gene (c.136C > T) established the genetic confirmation.

**Conclusion:**

This is the first case of Fabry disease reported in Sri Lanka. Awareness of specific clinical features aided clinical diagnosis long before access to genetic confirmation was available.

## Background

Fabry disease (FD), also known as Anderson-Fabry disease was first described by two independent dermatologists, Fabry in Germany and Anderson in England at the end of the nineteenth century, but it was not until the ‘70s that the deficient enzyme alpha-galactosidase A was discovered. This enzyme catalyzes the degradation of globotriaosylceramide (Gb3) to galactosylceramide, the lack of which, leads to the progressive and destructive accumulation of Gb3 in lysosomes. This accumulation histologically appears as zebra bodies or whorls which ultimately triggers a cascade of cytotoxic and inflammatory effects in affected tissues [1]. FD is a rare disease with a reported incidence of 1:117,000, however, it is likely to be underestimated due to missed or overlooked diagnoses [2]. Recent newborn screening studies have reported a higher incidence, indicating that it is more common than previously thought. Although excess Gb3 has accumulated by birth, most patients remain asymptomatic in the first decade, in contrast to other lysosomal storage diseases [3]. Specific clinical signs such as characteristic eye changes and angiokeratomas are useful to the clinician in making the diagnosis in suspected cases. We present a GLA gene mutation in a Sri Lankan boy with classic FD phenotype, which has been reported previously in a female and male with classic Fabry disease [4], in whom the diagnosis long preceded genetic confirmation followed by a discussion on the importance of eye findings and diagnostic markers and recent therapeutic advances of FD.

## Case presentation

A 13-year old boy, the second child to non-consanguineous healthy parents, presented following an afebrile convulsion with no prior history, with the seizure morphology being focal. He had an uneventful birth history and was developmentally normal, attending age-appropriate mainstream school. However, he was reported to have poor school performance by his parents.

Past medical history revealed episodes of burning sensation of the hands and feet over the past 2 years with each episode lasting between one to 3 days and originating from the palms and soles. Further inquiry revealed the absence of sweating in hot weather or post-exercise and he also gave a history of recurrent fever for which a cause could not be established. At this point, Fabry disease was suspected and a detailed neurological examination was performed, which identified unremarkable motor and cerebellar system examinations. Sensory examination revealed features of peripheral neuropathy, such as tactile hyperalgesia, compression hyperalgesia, dysesthesia, and hypohidrosis, but with normal lacrimation, salivation, temperature sensation, vibratory and proprioception sensation. Cranial nerve examination demonstrated nonspecific visual field defects in all four quadrants and slit-lamp examination revealed the presence of bilateral Cornea Verticillata. Despite these findings, he had no visual complaints and had an unremarkable past medical, ocular, and family history and had never been on any treatments that could cause corneal deposits, such as amiodarone, chloroquine, and indomethacin.

His sixteen-year-old brother has had similar complaint of intermittent acroparesthesia for which medical attention had not been sought as it had settled spontaneously with time.

Basic investigations including blood counts, urine analysis, serum electrolytes, renal and liver functions were found to be normal. He also had normal motor and sensory nerve conduction studies, hearing assessment, ultrasonically normal kidneys, structurally and functionally a normal heart, electroencephalogram, and brain imaging.

Diagnosis of Fabry Disease was confirmed by enzyme analysis via fluorometry, which revealed low Galactosidase A levels of 0.34 nmol/hr./ml (3–20 nmol/hr./ml). The *GLA* gene was analysed by next-generation sequencing of the coding region which revealed a missense mutation in Exon 1 of GLA, c.136C > T (p. His46Tyr) which was likely to be a pathogenic variant.

## Discussion and conclusions

In FD, two overlapping phenotypes are recognized; an early onset classical form with neuropathic pain, angiokeratoma, and hypohidrosis which precedes renal, cerebral and heart disease, and a later onset form with predominant manifestation in the heart [5]. These two phenotypes occur due to the variants of genetic mutation and it is the classic phenotype that presents itself in the paediatric population [5].

Classic Fabry disease seen in our patient, is the severe phenotype, having little or no functional alpha-galactosidase A enzyme activity (< 1%), usually manifesting by 10 years of age [6].

Initial manifestations apart from the acroparesthesia, hypohidrosis, pyrexia of unknown origin experienced in our patient include angiokeratomas (characteristic erythematous skin lesions, in the groin and hip areas) are often seen in the second decade. Cardiovascular disease, propensity for ischemic strokes, and renal disease become increasingly prominent by the third decade [5, 6]. With dialysis, renal transplant, and use of enzyme replacement therapy, the lifespan is now reported to exceed 50 years [7]. Later-onset variant is a mild form; it has 2–30% of residual enzyme activity and present between the third to the seventh decade, often diagnosed incidentally during the evaluation of unexplained heart failure, renal failure, or stroke [8].

The key to early and decisive diagnosis of our case was by the revelation of Cornea Verticillata (CV) or Vortex Keratopathy, which is whorl-like white to brown corneal opacities radiating to the periphery of the eye, best visualized by slit-lamp examination [9]. It is highly sensitive, as it is present in almost all affected males and more than 70% of heterozygous females and is also highly specific, only very rarely found in non-affected children. Therefore, the slit-lamp evaluation, which is non expensive, simple and non-invasive, available in even resource-limited settings is a very valuable ophthalmological tool for early diagnosis and can even be used for screening of female carriers [10]. Other rare differentials for this eye examination include the use of amiodarone, indomethacin, phenothiazines or undergoing radial keratotomy.

Data from the ongoing Fabry Outcome Survey has shown that CV occur in similar frequency in either sex, has been detected even in very young children (youngest 3 years old), has a higher prevalence in those with missense mutations of the GLA gene and its presence correlates with more severe disease [11, 12]. The only drawbacks are that CV are visually silent and therefore hardly sought medical attention. It requires evaluation by experienced ophthalmologists for correct identification [10]. Thus, recognizing the clinical importance of CV by Paediatric Ophthalmologists and Paediatricians might increase the number of early diagnoses made. Although CV is a valuable tool for early diagnosis and identification of patients at risk, it cannot be used to monitor disease progression or assess response to treatment [12].

In addition to CV, posterior spoke-like subcapsular cataracts called ‘Fabry cataract’ seen in 30% of the affected males and tortuous conjunctival or retinal vessels are two other important ocular signs aiding clinical diagnosis. Both these signs were absent in our patient [9].

The confirmatory workup for Fabry Disease includes detecting low levels of alpha galactosidase A and identifying specific *GLA* gene mutation [13]. Nerve conduction studies are usually normal, as it has low sensitively to detect small fibre neuropathy [14]. Urinary Gb3 is not a reliable marker as it is not elevated in late-onset disease or certain mutations in the *GLA* gene [13]. However, a novel diagnostic tool known as Lysosomal Gb3, which is found to be elevated in FD, has shown promising results in initial studies and can be used to classify classic and late-onset males and carrier females who may require treatment, who would otherwise be missed due to normal enzyme levels [15].

DNA sequencing is done on the *GLA* gene, which is mapped to the q22.1 region of the X chromosome with 7 exons distributed over 12,436 base pairs. Over 900 mutations are known so far, and it includes small deletions/insertions, large gene rearrangements, splicing defects, missense, and nonsense mutations [16]. The DNA sequencing in our patient identified a missense variant ‘H46Y’This mutation has led to the substitution of nucleotide cytosine by thymine at position 136 in the exon 1(c.136C > T) leading to the production of an abnormal protein, in which the amino acid histidine had been replaced by tyrosine at position 46 (p.His46Tyr). This change of *GLA* conformation and enzyme dysfunction is likely pathogenic and concluded that the patient had a variant form of FD. However, the exact manifestation of this new mutation is still unclear, thus further follow up and research is needed [4].

Early diagnosis is crucial for the early instauration of treatment. As a whole, caring for children with FD requires a multidisciplinary approach including lifelong supportive care, genetic counselling, advocating lifestyle modifications, long term monitoring of symptoms, prophylactic medications, and screening other family members. Gabapentin, amitriptyline, and carbamazepine have found to be beneficial in the treatment of chronic neuropathic pain. Patients may also benefit from avoidance of triggers such as significant physical activity and extreme weather changes [13].

The initiation of definitive treatment is based on a confirmed pathogenic mutation, low levels of GLA enzyme, typical clinical manifestations, with Lysosomal Gb3 providing supporting evidence of disease activity. Therapy is aimed at increasing functional enzyme levels or reduction in the accumulation of Gb3 and include enzyme and non-enzyme-based therapies. Enzyme replacement therapy (ERT) remains the mainstay of treatment. However, lifelong two weekly infusions in paediatric patients have proven to be very challenging due to cost, difficult venous access, and risk of infusion-related reactions. Pegunigalsidase α an investigational ERT which is less immunogenic with a longer half-life and α-GAL mRNA which stimulates α-GLA production are undergoing clinical trials. Gene therapy via ex vivo (hemopoietic stem cells harvested from the patient are reinserted after gene editing) and in vivo (direct infusion of viral vectors for gene transduction) methods are also in the pipeline with the promise of a potential cure [17].

Non-enzyme-based therapies include Migalastat, an oral chaperone therapy that works on patients with amenable missense mutations of the *GLA* gene that produces a strain of mutant enzymes with residual activity. It works by improving protein folding and trafficking of the defective enzyme into the lysosome, the site of enzymatic activity, and thereby rescuing it from premature degradation. Migalastat is orally available with every other day dosing and can cross the blood-brain barrier as well. The variant reported in our patient was found to be not amenable to treatment with Migalastat. Substrate reduction therapy is another novel non-enzyme-based therapy undergoing clinical trials and it works by limiting the production of Gb3 from ceramide [17].

In conclusion, this is the first case of Fabry disease and the first mutation of *GLA* gene reported in Sri Lanka. Only a few cases are reported from the Asian subcontinent.

Our case report highlights the importance of history, clinical examination and a simple slit lamp examination for a confident clinical diagnosis of FD, even in the absence of targeted confirmatory enzyme assays and genetic analysis.

## Data Availability

Not applicable.

## Abbreviations

FD: Fabry Disease
CV: Cornea Verticillata
GLA: Galactosidase Alpha
Gb3: Globotriaosylceramide
ERT: Enzyme Replacement Therapy
